# Identification of three T cell-related genes as diagnostic and prognostic biomarkers for triple-negative breast cancer and exploration of potential mechanisms

**DOI:** 10.3389/fgene.2025.1584334

**Published:** 2025-06-18

**Authors:** Zhi-Chuan He, Zheng-Zheng Song, Zhe Wu, Peng-Fei Lin, Xin-Xing Wang

**Affiliations:** ^1^ Department of Thyroid and Breast Surgery, The First Hospital of Putian City, Putian, Fujian, China; ^2^ Department of Breast and Thyroid Surgery, Yidu Central Hospital of Weifang, Qingzhou, Shandong, China; ^3^ Department of Pathology, The First Hospital of Putian City, Putian, Fujian, China

**Keywords:** Triple-negative breast cancer, T cell-related genes, diagnosis, prognosis, immune infiltration

## Abstract

**Background:**

Triple-negative breast cancer (TNBC) is an aggressive subtype of breast cancer (BRCA) with limited therapeutic targets. This study aimed to identify T cell-related signatures for TNBC diagnosis and prognosis.

**Methods:**

Clinical data and transcriptomic profiles were obtained from the TCGA-BRCA dataset, and single-cell RNA sequencing (scRNA-seq) data were downloaded from the GEO database. Differentially expressed genes (DEGs) between TNBC and other BRCA subtypes were intersected with T cell-related genes to identify candidate biomarkers. Machine learning algorithms were used to screen for key hub genes, which were then used to construct a logistic regression (LR) model. Immune cell infiltration patterns were analyzed between high- and low-LR score groups, and Kaplan–Meier analysis evaluated the prognostic significance of hub genes. Functional enrichment and pathway analysis were performed using GSEA, and scRNA-seq data further explored hub gene-related pathways in immune cells.

**Results:**

Three hub genes (*CACNA1H*, *KCNJ11*, and *S100B*) were identified with strong diagnostic and prognostic relevance in TNBC. The LR model based on these genes achieved an AUC of 0.917 in diagnosing TNBC from other BRCA subtypes. Low LR scores were associated with poorer overall survival and reduced immune cell infiltration, particularly CD8 T cells and cytotoxic lymphocytes. *S100B* showed strong associations with the cytokine–cytokine receptor interaction pathway, JAK–STAT signaling, and T cell receptor signaling.

**Conclusion:**

*CACNA1H*, *KCNJ11*, and *S100B* are potential diagnostic and prognostic biomarkers in TNBC. Their immune-related functions highlight their potential for guiding targeted immunotherapy strategies.

## 1 Introduction

Triple-negative breast cancer (TNBC) is a highly aggressive subtype of breast cancer (BRCA) characterized by the absence of estrogen receptor (ER), progesterone receptor (PR), and human epidermal growth factor receptor 2 (HER2) expression (Aysola et al., 2013). It accounts for approximately 15%–20% of all BRCA cases, disproportionately affecting younger women and those of African descent (Xiong et al., 2024; Dietze et al., 2015). TNBC is characterized by rapid progression, early metastasis, and poor prognosis compared to other BRCA subtypes (Manjunath and Choudhary, 2021). For non-metastatic BRCA, surgical intervention is the primary treatment method, while hormone receptor-positive BRCA is mainly treated through endocrine therapy (Pan et al., 2023). TNBC, due to the absence of targeted therapies, has a more complex treatment strategy. Chemotherapy is the main treatment option for TNBC, but the efficacy of chemotherapy is often unsatisfactory (Pan et al., 2023; Dobovisek et al., 2024). Immunotherapy is a novel promising option for the treatment of TNBC, however, the clinical response rate of immune checkpoint inhibitors (ICIs) as a single therapy is relatively low (Rahman et al., 2023).

Snowballing research suggests that the unique immunosuppressive tumor microenvironment (TME) of TNBC is associated with tumor therapeutic failure (Zhang et al., 2024). TME of solid cancers mainly has three immune-related phenotypes: immune-inflamed (abundant T cell infiltration into the tumor), immune-excluded (abundant T cell infiltration but trapped in the stroma surrounding the cancer nest), and immune-desert (scarce T cell infiltration into the tumor) (Wu et al., 2022). T cells, including CD4 and CD8 T cells, as part of the TME, participate in the recognition and elimination of tumor cells. T cell-mediated anti-tumor immune responses form the basis of tumor immunotherapy (Yan et al., 2022), and T cell characteristics have been developed for predicting cancer prognosis and immune therapy responses (Liu et al., 2024). A study has shown that CD4^+^ T cells in peripheral blood can stably predict all clinical outcomes for TNBC patients (Li et al., 2022). We speculate that identifying T cell-related genes as diagnostic biomarkers for TNBC could enable early detection, thereby providing significant clinical value.

In this study, we identified T cell-related genes that could serve as diagnostic biomarkers for TNBC using bioinformatics and explored the association of these genes with the tumor immune microenvironment (TIME) and their potential action mechanisms. This research aimed to provide new perspectives on the role of T cell-related genes in the progression and treatment of TNBC. Furthermore, investigating the intrinsic mechanisms of these genes may reveal new therapeutic targets and strategies, thereby enhancing the efficacy of immunotherapy in TNBC and ultimately improving patient outcomes.

## 2 Methods

### 2.1 Data acquisition

The RNA-seq data and clinical information for BRCA patients were obtained from The Cancer Genome Atlas (TCGA) database (https://tcga-data.nci.nih.gov/tcga/), including 123 TNBC samples, 979 other BRCA subtype samples, and 114 normal samples. The baseline clinical information is shown in Supplementary Table S1. An external validation dataset GSE58812 (including 107 TNBC tissue samples) with survival data and expression profiles, as well as the single-cell RNA sequencing (scRNA-seq) dataset GSE176078 (including 10 TNBC samples and 16 other BRCA subtype samples), were acquired from the Gene Expression Omnibus (GEO) database (http://www.ncbi.nlm.nih.gov/geo/). T cell-related genes were searched from the GeneCards database (https://www.genecards.org) by searching with the keyword “T cell” on 26 August 2024. GeneCards integrates data from multiple biological databases and provides a comprehensive, evidence-weighted view of gene-function relationships (Stelzer et al., 2016). The relevance score serves as a useful filter for related signature genes with strong cumulative evidence. In this study, genes with a relevance score ≥10 were retained to ensure a strong association with T cell biology. A total of 6,615 genes were included in the final gene set used for downstream analysis (Supplementary Table S2).

### 2.2 Differential gene expression analysis and functional enrichment analysis

The Limma package in R was used to identify differentially expressed genes (DEGs) between the TNBC and other subtypes. Transcript-level expression data (TPM) were downloaded from the TCGA-BRCA cohort. To prepare the data for linear modeling, we applied a log2 transformation with a pseudo count [log2 (TPM +1)] to stabilize the variance across genes. Low-expression genes (mean TPM <1 across all samples) were filtered out before analysis to reduce background noise. The genes with p < 0.05 and |log2FC| > 1 were considered DEGs, and results were visualized using a volcano plot. These DEGs were then intersected with T cell-related genes through a Venn diagram. Gene Ontology (GO) and Kyoto Encyclopedia of Genes and Genomes (KEGG) enrichment analyses were performed to reveal the overlapping genes’ functions using R packages “clusterProfiler”, and results were visualized through “Goplot” package.

### 2.3 Identification of hub genes using machine learning algorithms

To identify T cell-related prognostic genes, the least absolute shrinkage and selection operator (Lasso) regression analysis was first applied on the overlapping genes using the “glmnet” R package. The regularization parameter lambda was selected based on 10-fold cross-validation using the cv.glmnet function, and the lambda value corresponding to the minimum mean cross-validated error (lambda.min) was chosen. The alpha parameter was set to 1, which corresponds to standard Lasso regression. Subsequently, we utilized three machine learning algorithms—random forest (RF) (Rigatti, 2017), XGBoost (Li et al., 2019), and AdaBoost (Sorayaie Azar et al., 2022)—to rank the importance of prognosis-related genes identified by Lasso. RF was implemented using the “randomForest” R package with ntree = 500 and mtry = sqrt(p) (where p is the number of input features). Genes were ranked based on the MeanDecreaseGini index. XGBoost was performed using the “xgboost” R package with parameters nrounds = 100, eta = 0.3, max_depth = 6, and objective = “binary: logistic”. Feature importance was ranked using the “gain” metric. AdaBoost was conducted using the “adabag” R package with default parameters (mfinal = 50). Gene importance was ranked based on the decrease in classification error. For each algorithm, the top 10 genes were visualized using bar plots generated via the “ggplot2” package in R. Finally, the intersection of the top 5 genes identified by all three algorithms was defined as the hub genes for further analysis.

### 2.4 Construction and evaluation of a logistic regression (LR) model

Based on the hub genes, a diagnostic model for TNBC was established using the LR analysis. The LR score for each TNBC patient in the TCGA-BRCA cohort was calculated using expression levels and regression coefficients of hub genes in the LR analysis. The model’s clinical value was evaluated through receiver operating characteristic (ROC) curves and decision curve analysis (DCA) curves. pROC package and ggDCA package were used to create ROC curves and DCA curves, respectively. Then, TNBC samples were divided into high and low groups based on the optimal truncation value of the LR score. Kaplan–Meier (K-M) survival curves were generated using the survival package to reveal the overall survival (OS) between the two groups. To further evaluate the prognostic utility of the LR model, we applied it to the external validation dataset GSE58812. As described above, the LR score for each TNBC patient in GSE58812 was calculated based on the expression levels of the hub genes and the corresponding regression coefficients. Patients were then divided into high and low LR score groups using the optimal cutoff value. Subsequently, K-M analysis was performed to compare OS between the two groups.

Additionally, LR score differences in different clinical subgroups (such as age, TNM stages, and pathological) were analyzed.

### 2.5 Tumor immune microenvironment (TIME) analysis

Immune cell infiltration scores and the tumor immune dysfunction and exclusion (TIDE) scores between low and high LR score groups were evaluated using the MCPcounter package and TIDE software (http://tide.dfci.harvard.edu/), respectively. Higher TIDE score means a greater possibility of immune escape.

### 2.6 Role of hub genes in TNBC

K-M curves were utilized to compare OS between groups with low and high expression levels of hub genes. The log-rank test was used to assess statistical significance. To provide a more robust estimate of survival differences and avoid the proportional hazards assumption, we performed restricted mean survival time (RMST) analysis, with the truncation time point (τ) set to 23 years. To further explore the subtype specificity of the hub genes, we compared their expression levels in TNBC versus non-TNBC samples from the TCGA-BRCA cohort using the Wilcoxon rank-sum test. All statistical analyses and visualizations were conducted in R, with survival, survminer, and survRM2 packages being used.

Gene Set Enrichment Analysis (GSEA) can be used to assess whether a predefined set of genes shows statistically significant differences between two biological states (Lu et al., 2024). To explore the biological pathways associated with each hub gene, GSEA was performed using transcriptomic data from the TCGA-BRCA cohort. For each hub gene, patients were divided into high- and low-expression groups based on the median expression value. GSEA was conducted using the “clusterProfiler” R package, where all genes were ranked according to their differential expression between the two groups. Pathways with a false discovery rate (FDR) < 0.025 and p < 0.05 were considered significantly enriched.

### 2.7 Correlation of hub genes with immune cells

The MCPcounter algorithm was used for immune infiltration analysis to quantify the immune cell abundance for BRCA samples. It can quantify the absolute abundance of eight immune cells (B-cell lineage, CD8 T cells, cytotoxic lymphocytes, monocytic lineage, myeloid dendritic cells, natural killer (NK) cells, neutrophils, and T cells) and two stromal cells (fibroblasts and endothelial cells) using transcriptome data (Zheng et al., 2022). Differences in cell abundance between the high and low LR score groups were assessed using Student’s t-test. Spearman correlation coefficients were calculated to evaluate the associations between the expression of each hub gene and the estimated abundance of each immune/stromal cell type. Correlation significance was assessed using two-tailed p-values, and results with p < 0.05 were considered statistically significant. The correlations were visualized using a lollipop plot constructed with the “ggplot2” R package.

### 2.8 scRNA-seq analysis

Preprocessing and filtering of scRNA-seq data were performed using the Seurat package. The quality control criteria were set as nFeature_RNA >500, 1,000 < nCount_RNA <20,000, and percent.mt < 20. After standardizing the data using the scaling function, principal component analysis was conducted to identify significant principal components. Subsequently, t-distributed stochastic neighbor embedding (t-SNE) analysis was performed to identify cell clusters. These cell clusters were annotated using SingleR version 2.0.0 in R. The Wilcoxon-Mann-Whitney test was used to calculate the expression differences of each gene across different samples in the model. Additionally, pathway scores for three pathways identified in the GSEA were calculated in the annotated cells using the singScore function in Seurat. “singscore” quantifies the activity level of a specific biological function or process within a single sample or cell (Zhao et al., 2024). Spearman correlation analysis was performed using the cor.test function in R to investigate the associations between these three pathways and three hub genes. The results were visualized using ggplot2 with scatter plots.

### 2.9 Statistical analysis

R software version 4.1.2 was used for statistical analysis, and p < 0.05 was considered statistically significant.

## 3 Results

### 3.1 Identification of T cell-related DEGs in TNBC

**FIGURE 1:**
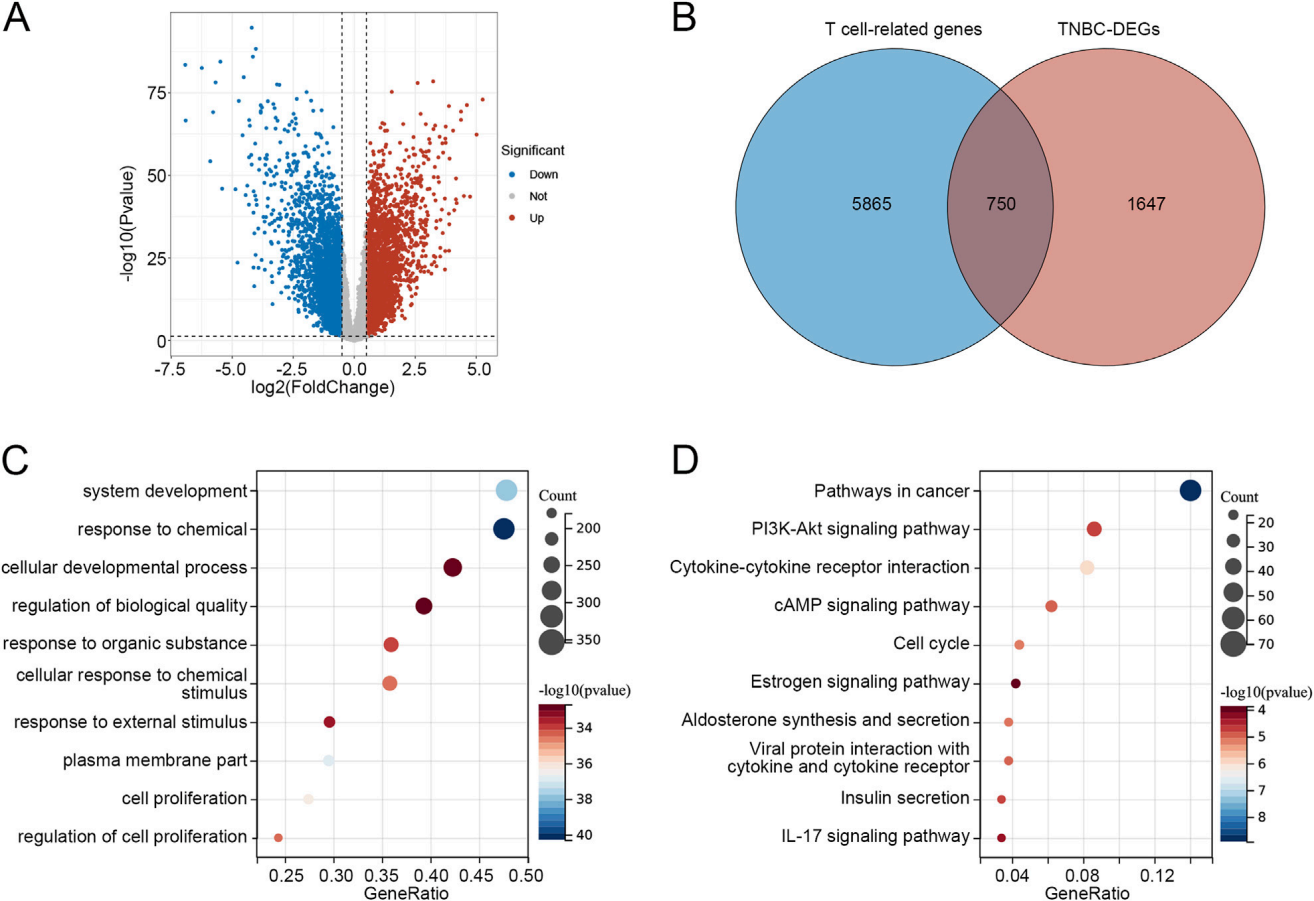
Identification of T cell-related DEGs in TNBC **(A)** Volcano plot of DEGs between TNBC and other BRCA subtypes. **(B)** Venn diagram identified 750 overlapping genes between T cell-related genes and TNBC-DEGs. **(C)** Go enrichment analysis of 750 overlapping genes. **(D)** KEGG pathways related to 750 overlapping genes. Abbreviations: DEGs, differentially expressed genes; TNBC, triple-negative breast cancer; BRCA, breast cancer; GO, Gene Ontology; KEGG, Kyoto Encyclopedia of Genes and Genomes.

As shown in the volcano plot (Figure 1A), a total of 2,397 DEGs were identified between TNBC and other subtypes of BRCA. Then, both upregulated and downregulated DEGs were intersected with T cell-related genes to comprehensively capture T cell-related dysregulation patterns associated with TNBC, resulting in 750 overlapping genes (Figure 1B; Supplementary Table S3). GO enrichment analysis revealed that the 750 genes were correlated to functions such as system development, response to chemical, cellular developmental process, and regulation of biological quality (Figure 1C). The KEGG pathways related to these 750 overlapping genes were enriched in the PI3K-Akt signaling pathway, cytokine-cytokine receptor interaction, estrogen signaling pathway, and IL-17 signaling pathway (Figure 1D).

### 3.2 Identification of hub genes related to prognosis in TNBC

**FIGURE 2:**
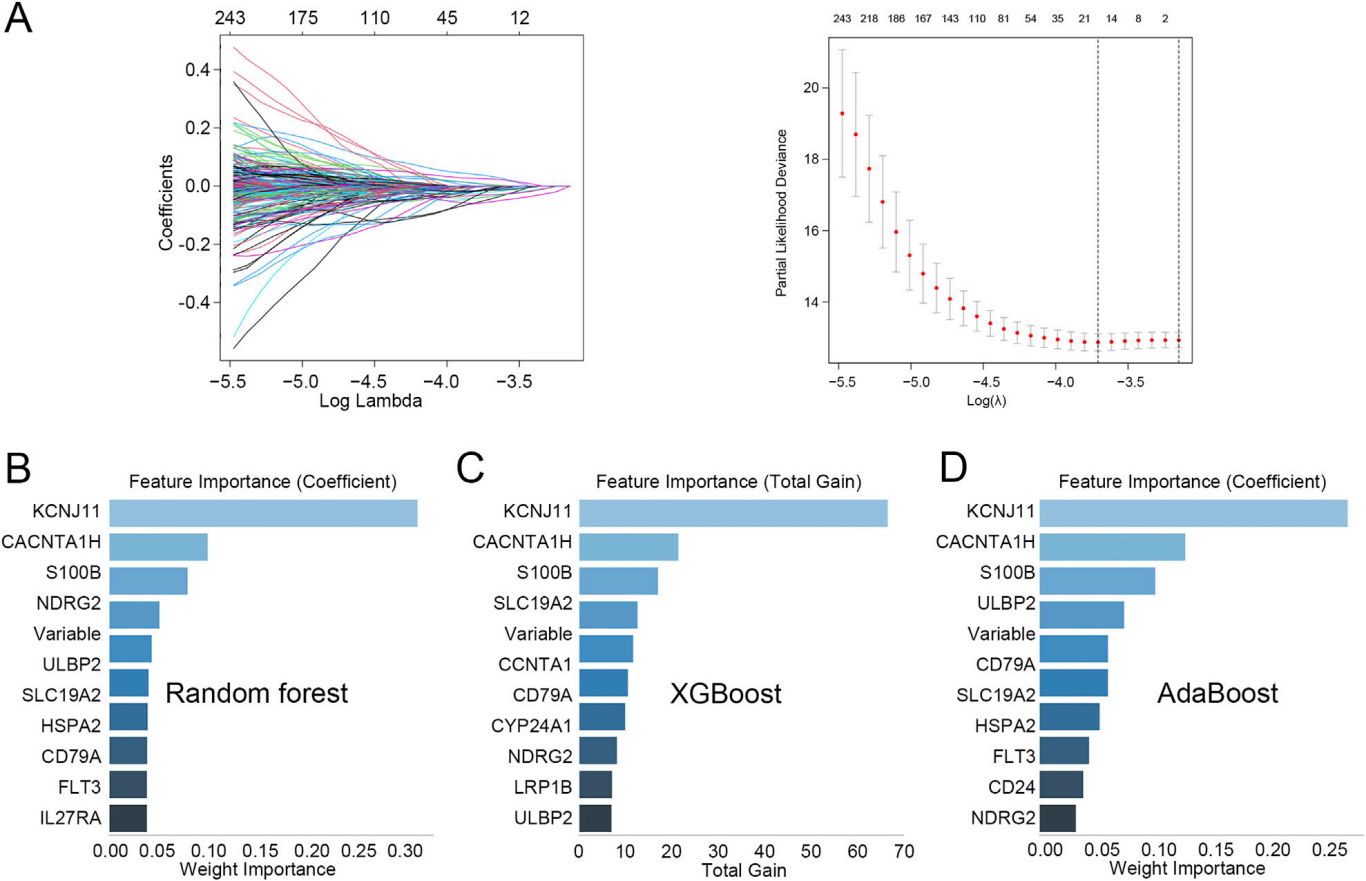
Identification of hub genes related to prognosis in TNBC **(A)** Lasso regression analysis was performed on the 750 T cell-related DEGs. **(B–D)** The top 10 genes identified through **(B)** random forest, **(C)** XGBoost, and **(D)** AdaBoost.

Lasso regression analysis was performed on the 750 T cell-related DEGs to obtain genes related to the prognosis of TNBC, and a total of 17 genes were identified (Figure 2A). Subsequently, according to importance, these 17 genes were ranked using three machine learning algorithms RF, XGBoost, and AdaBoost. The top 10 genes selected by RF, XGBoost, and AdaBoost were shown in Figures 2B–D, respectively. After intersecting the top five genes selected by these three machine learning algorithms, three hub genes were acquired, including *CACNA1H*, *KCNJ11*, and *S100B*.

### 3.3 Construction of an LR model

Binary LR analysis was performed by integrating three identified hub genes *CACNA1H*, *KCNJ11*, and *S100B* as independent variables, and TNBC as the dependent variable. As shown in Table 1, *CACNA1H*, *KCNJ11*, and *S100B* were independent predictors for TNBC (p < 0.05). Based on the multivariable analysis results, an LR model was constructed with an LR score computed: 0.5000*CACNA1H – 0.499*KCNJ11 + 0.252*S100B.

**TABLE 1:** Logistic regression analysis based on three hub genes.

Genes	Coefficients	Odds ratio (95% confidence interval)	p-value
CACNA1H	−0.500	0.607 (0.517–0.712)	< 0.001
KCNJ11	−0.499	0.607 (0.530–0.695)	< 0.001
S100B	0.252	1.286 (0.160–1.426)	< 0.001

### 3.4 Clinical relevance of the LR model

**FIGURE 3:**
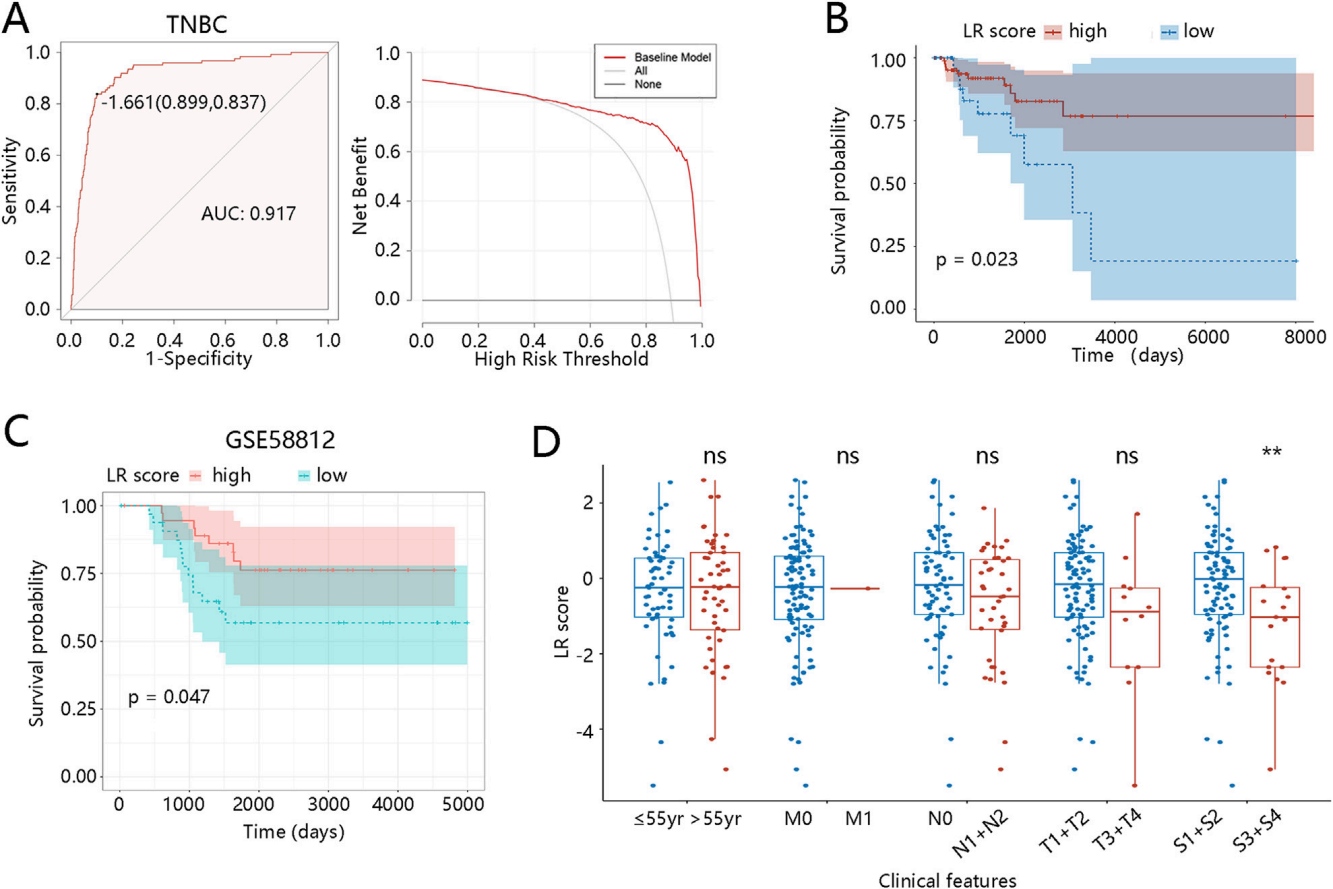
Clinical relevance of the LR model in TNBC patients **(A)** ROC and DCA curves for diagnosing TNBC. **(B)** Kaplan-Meier curve for the LR model based on the TCGA dataset. **(C)** Kaplan-Meier curve for the LR model based on the GSE58812. **(D)** LR score differences between different clinical subgroups. **p < 0.01, ns means no significance, yr means year. Abbreviations: LR, logistic regression; TNBC, triple-negative breast cancer; ROC, receiver operator characteristic; AUC, area under curve; DCA, decision curve analysis.

The ROC analysis was then conducted to evaluate the diagnostic value of the LR model in distinguishing TNBC from other subtypes with an AUC value of 0.917 (Figure 3A). When the threshold was about >0.5, there was a clinical net benefit for the LR model (Figure 3A). Moreover, the ROC analysis was also conducted to analyze the role of the LR model in distinguishing BRCA from normal groups (AUC = 0.846), indicating satisfactory diagnostic performance (Supplementary Figure S1A). The DCA result is also shown in Supplementary Figure S1A.

Subsequently, the K-M analysis was used to explore the association of the LR model with the prognosis of TNBC patients. Individuals with low LR scores had significantly shorter OS than those with high scores (p = 0.023, Figure 3B), and this finding was validated in the external dataset (p = 0.047, Figure 3C). Additionally, K-M curves revealed the correlation between the LR model and the prognosis of BRCA patients. As shown in Supplementary Figure S1B, BRCA patients with low scores had worse prognoses than those with high scores (p = 0.0058). To further explore the clinical relevance of the LR score, we assessed its distribution across subgroups with different clinicopathological features, including age, TNM stage, and pathological stage. As shown in Figure 3D, LR scores in the TNBC patients at S3 + S4 stages were significantly lower (p < 0.01). In BRCA patients, those over 55 years old and at N1 + N2/S3 + S4 stages also displayed lower LR scores (p < 0.05) (Supplementary Figure S1C). These observations reveal that lower LR scores are enriched in patients with more advanced clinical stages, suggesting that the LR score may reflect tumor progression and aggressiveness. Collectively, these results support the prognostic value and potential clinical applicability of the LR model.

### 3.5 TME landscapes in two different groups

**FIGURE 4:**
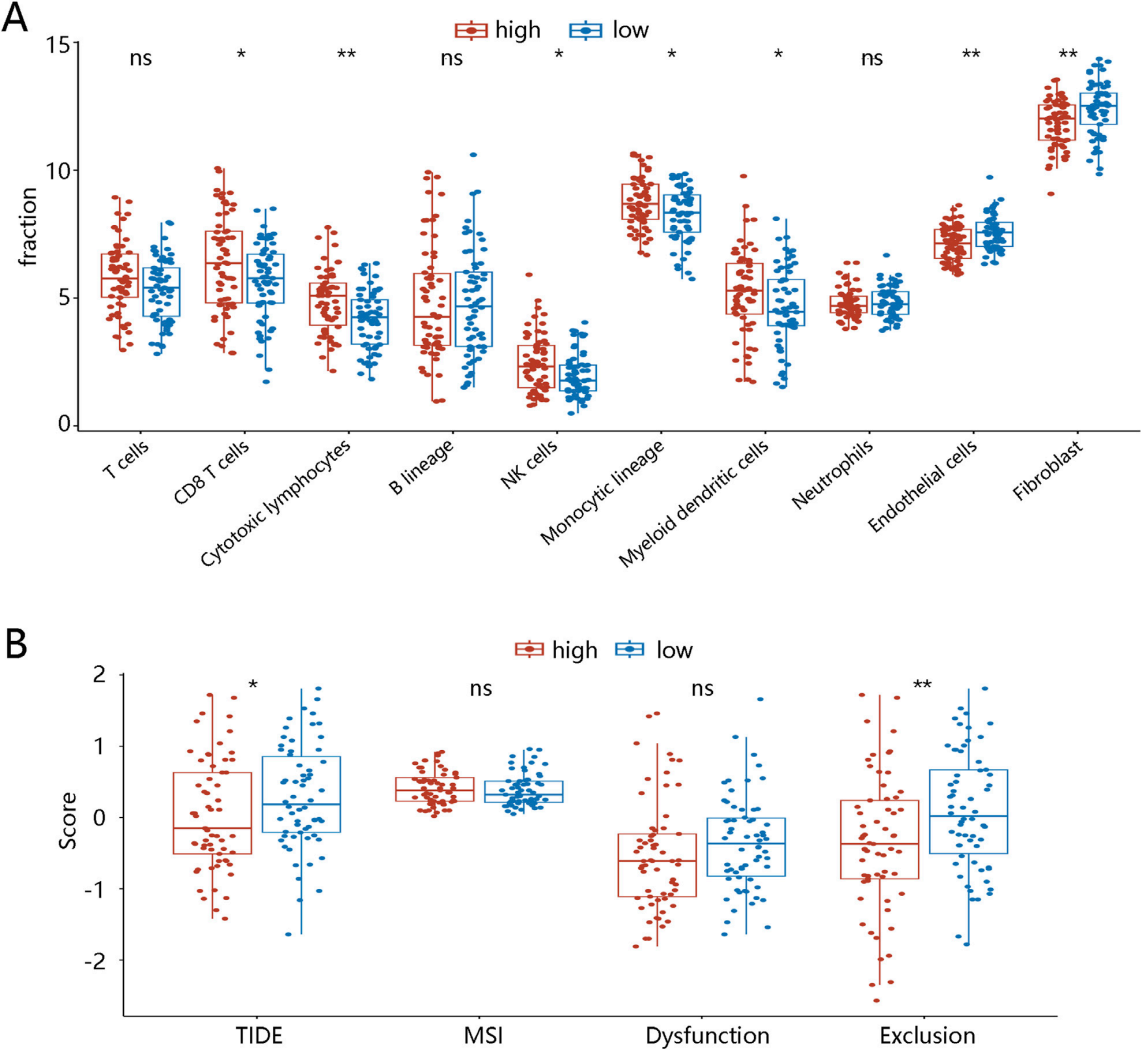
Tumor microenvironment landscapes between two LR score groups in TNBC patients **(A)** Immune cell infiltration in high- and low-LR score groups. **(B)** TIDE in high- and low-diagnostic score groups. *p < 0.05, ***p < 0.001, ****p < 0.0001, ns means no significance. Abbreviations: LR, logistic regression; TNBC, triple-negative breast cancer; NK, natural killer; TIDE, tumor immune dysfunction and exclusion; MSI, microsatellite instability.

Cell infiltration was then analyzed to explore the TME landscapes of TNBC patients between different LR score groups. Levels of CD8 T cells, cytotoxic lymphocytes, NK cells, monocytic lineage, and myeloid dendritic cells were significantly lower in the low LR score group than in the high LR score group. Conversely, levels of endothelial cells and fibroblasts were significantly higher in the low LR score group (Figure 4A). To further reveal the role of the LR model in immune therapy in TNBC patients, we performed the TIDE analysis. As illustrated in Figure 4B, the TIDE and exclusion scores were higher in the low LR score group (p < 0.05), although microsatellite instability (MSI) and dysfunction scores showed no significant differences. These results suggest that TNBC patients with low LR scores may be in an immunosuppressed microenvironment, potentially increasing their likelihood of immune escape. We also investigate the TME landscape of BRCA patients. Immune infiltration analysis using the MCPcounter algorithm showed that, except for fibroblast, the levels of the other 9 cell types were significantly lower in the low LR score groups compared with those in the high LR score groups (p < 0.05, Supplementary Figure S2A). In comparison to the high LR score group, the exclusion score was significantly higher in the low LR score group (p < 0.001), while TIDE, dysfunction, and MSI scores showed no significant differences between the two groups (Supplementary Figure S2B).

### 3.6 Prognosis performance and expression of three hub genes in TNBC

**FIGURE 5:**
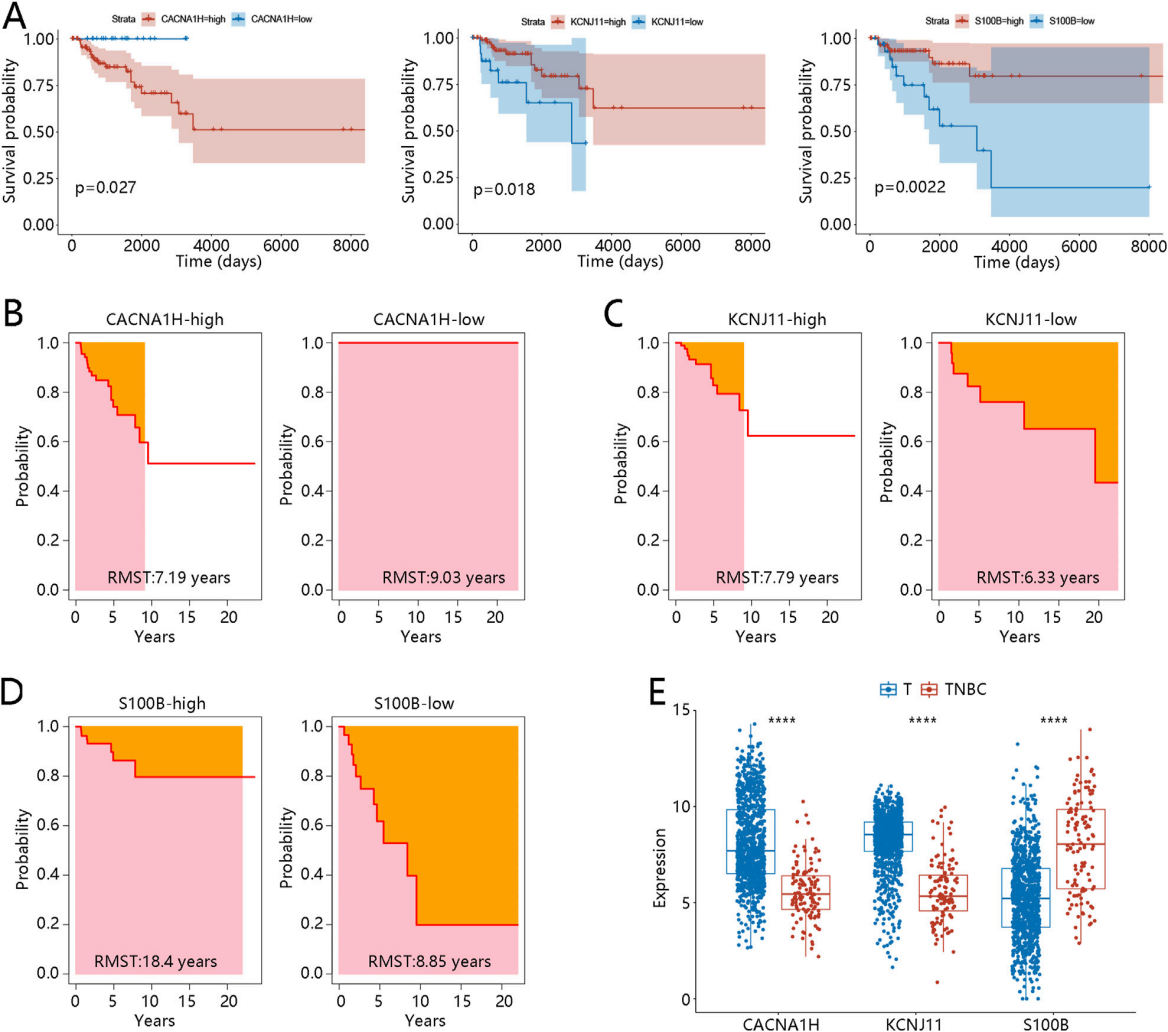
Prognosis performance expression of three hub genes in TNBC **(A)** Kaplan-Meier curves of three hub genes *CACNA1H*, *KCNJ11*, and *S100B*. **(B-C)** RMST analysis for three hub genes **(B)** *CACNA1H*, **(C)** *KCNJ11*, and **(D)** *S100B*. **(E)** Expression of three hub genes between other BRCA subtype samples (defined as T) and TNBC samples; ****p < 0.0001. Abbreviations: TNBC, triple-negative breast cancer; BRCA, breast cancer; RMST, restricted mean survival time.

Moreover, K-M curves were utilized to reveal the prognosis value of three hub genes in TNBC. The results showed that patients with high expression of *CACNA1H* or low expression of *KCNJ11* and *S100B* had significantly worse prognosis (p < 0.05, Figure 5A). RMST analysis further validated these results, as shown in Figures 5B–D. The mean survival time of patients with high expression of *CACNA1H* was 7.19 years, while that of the low-expression group was 9.03 years, with a significant difference (p = 0.01, Table 2). For *KCNJ11* and *S100B*, patients with low expression had mean survival times of 6.33 and 8.85 years, respectively, which were significantly shorter than those of the high-expression groups (p < 0.05, Table 2). Expression analysis revealed that, compared to other BRCA subtypes, CACNA1H and KCNJ11 were significantly downregulated in TNBC, whereas S100B was significantly upregulated (p < 0.0001, Figure 5E).

**TABLE 2:** RMST of TNBC patients in different hub gene expression groups.

	High-RMST (year)	Low-RMST (year)	Differences (95%CI)	p
CACNA1H	7.19	9.03	−1.84 (−3.22, −0.42)	0.01
KCNJ11	7.79	6.33	1.46 (0.16, 2.78)	0.03
S100B	18.40	8.85	9.55 (8.13, 10.77)	< 0.01

To further investigate the prognostic value of these three hub genes, survival analysis was performed using all tumor samples from the TCGA-BRCA dataset. As shown in Supplementary Figure S3A, patients with high expression of *CACNA1H* or low expression of *S100B* had shorter OS (p < 0.01). However, the K-M curve for *KCNJ11* showed a crossing point around 4,000 days, prompting us to conduct RMST analysis. The results for *CACNA1H* and *S100B* were consistent with the K-M analysis, showing that the high expression group of *CACNA1H* had a shorter survival time (Supplementary Figure S3B), while the low expression group of *S100B* had a shorter survival time (Supplementary Figure S3D) (Supplementary Table S4). For *KCNJ11*, RMST analysis showed that the low expression group had a shorter survival time, indicating a worse prognosis (Supplementary Figure S3C). These results were consistent with finding in the TNBC patients. However, compared with normal tissue samples, CACNA1H and KCNJ11 were significantly upregulated in BRCA, whereas S100B was significantly downregulated (p < 0.0001, Supplementary Figure S3E). Although the expression patterns of these hub genes vary across BRCA subtypes and normal tissues, their distinctive expression trends and prognostic associations in TNBC suggest that they may play crucial roles in TNBC progression and immune microenvironment modulation.

### 3.7 Signaling pathways and immune cells related to three hub genes

**FIGURE 6:**
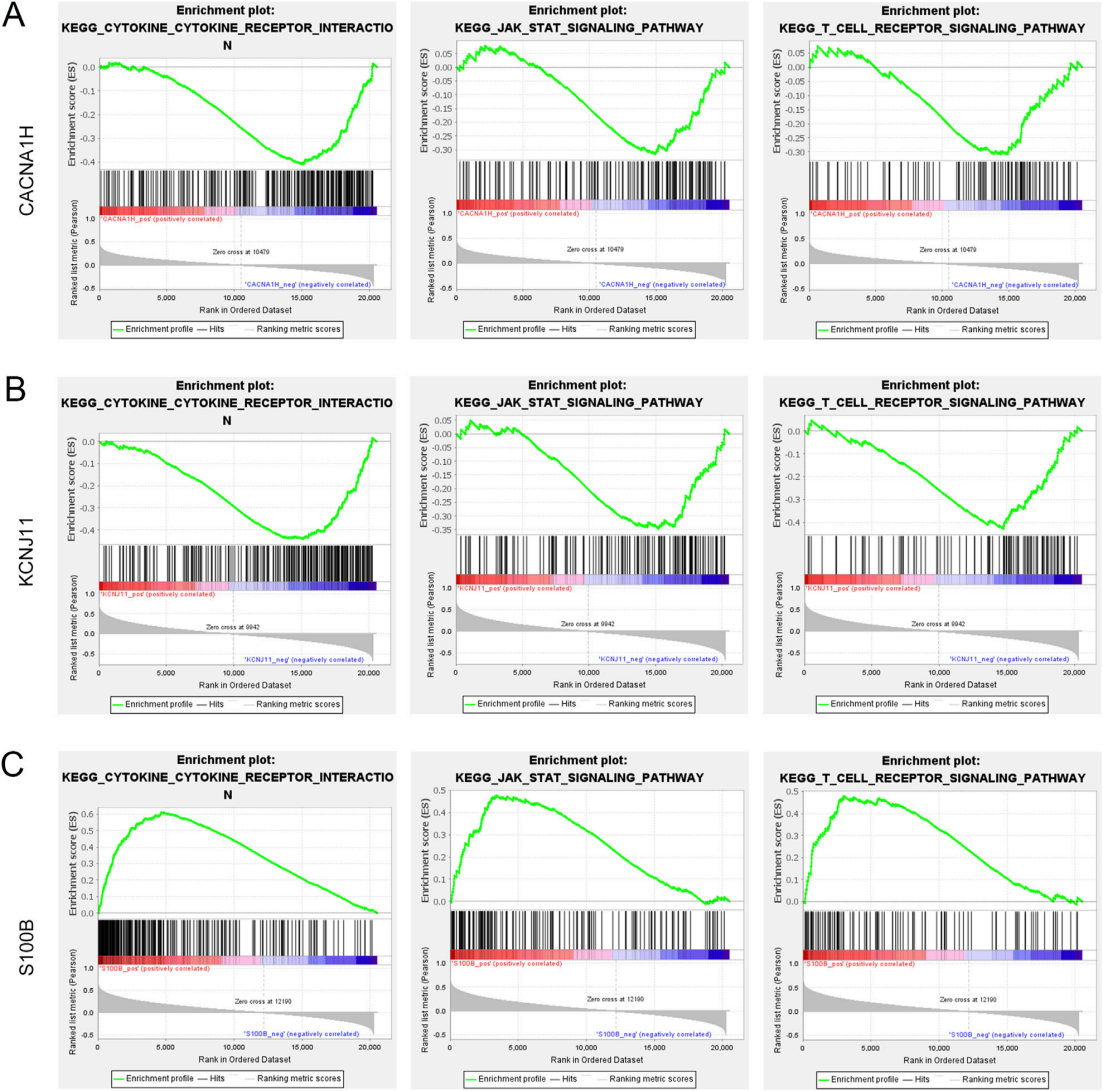
Gene set enrichment analysis of three hub genes **(A–C)**. Three pathways (cytokine-cytokine receptor interaction pathway, JAK-STAT signaling pathway, and T cell receptor signaling pathway) were related to **(A)** *CACNA1H*, **(B)** *KCNJ11*, and **(C)** *S100B*.

GSEA was performed to further explore the functions of three hub genes in TNBC. Three TME-related pathways associated with all hub genes were enriched, including the cytokine-cytokine receptor interaction pathway, JAK-STAT signaling pathway, and T cell receptor signaling pathway (Figures 6A–C). *CACNA1H* and *KCNJ11* were negatively correlated to these three pathways, while *S100B* was positively related to these pathways. In addition, the three hub genes were significantly correlated to most of the 10 immune cells identified above.

**FIGURE 7:**
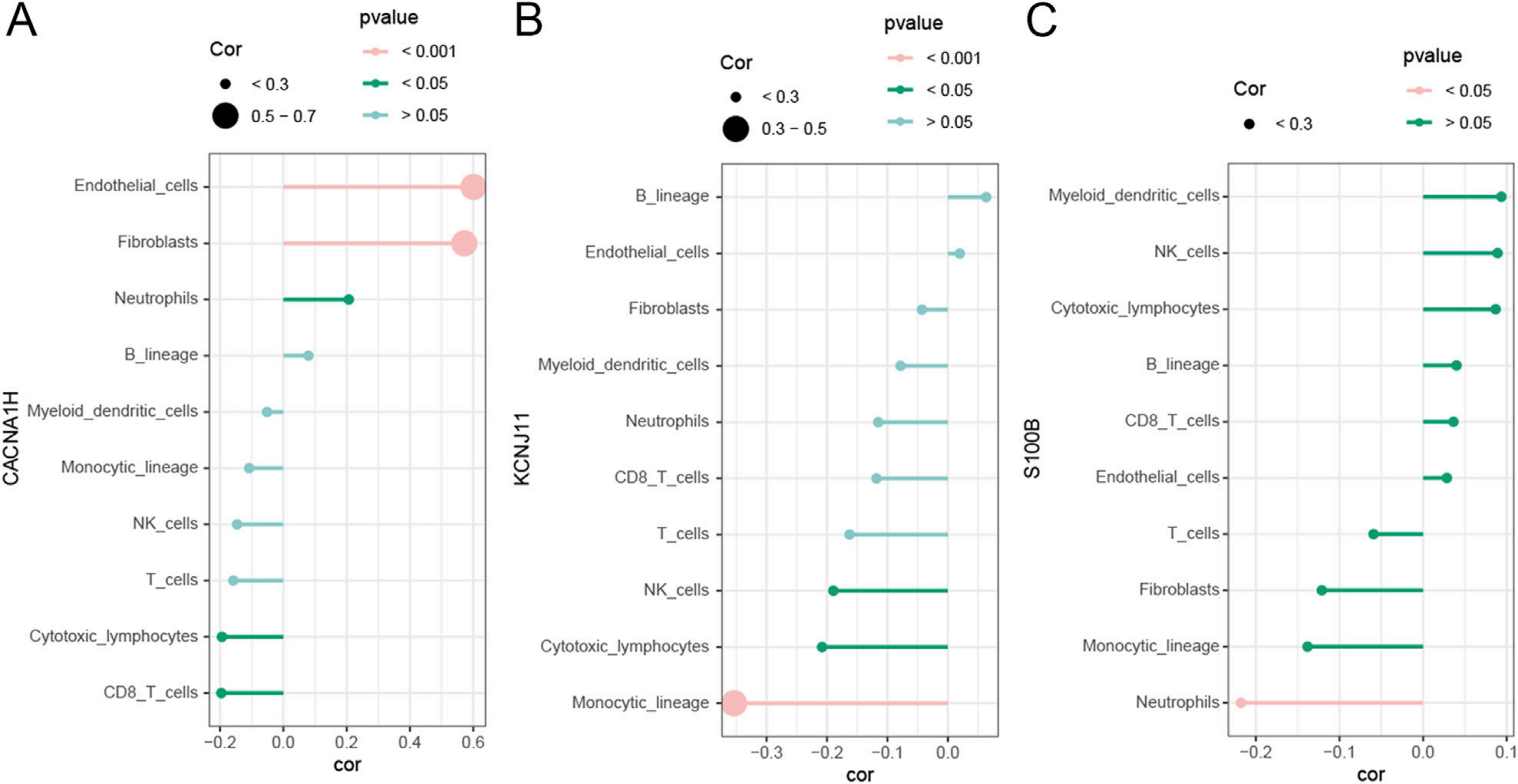
Correlation of three hub genes with immune cells **(A–C)**. Correlation of immune cells with **(A)** *CACNA1H*, **(B)** *KCNJ11*, and **(C)** *S100B*.

Because of the enrichment of TME-related pathways, we further explore the association of hub genes with 10 cell types identified in the MCPcounter algorithm. As shown in Figure 7A, *CANA1H* was positively related to fibroblasts, endothelial cells, and neutrophils while negatively related to cytotoxic lymphocytes and CD8 T cells. *KCNJ11* was negatively correlated to NK cells, cytotoxic lymphocytes, and monocytic lineage (Figure 7B). *S100B* was positively associated with myeloid dendritic cells, CD8 T cells, NK cells, and cytotoxic lymphocytes (Figure 7C).

### 3.8 Immune cell distribution and hub gene-related pathways in TNBC

**FIGURE 8:**
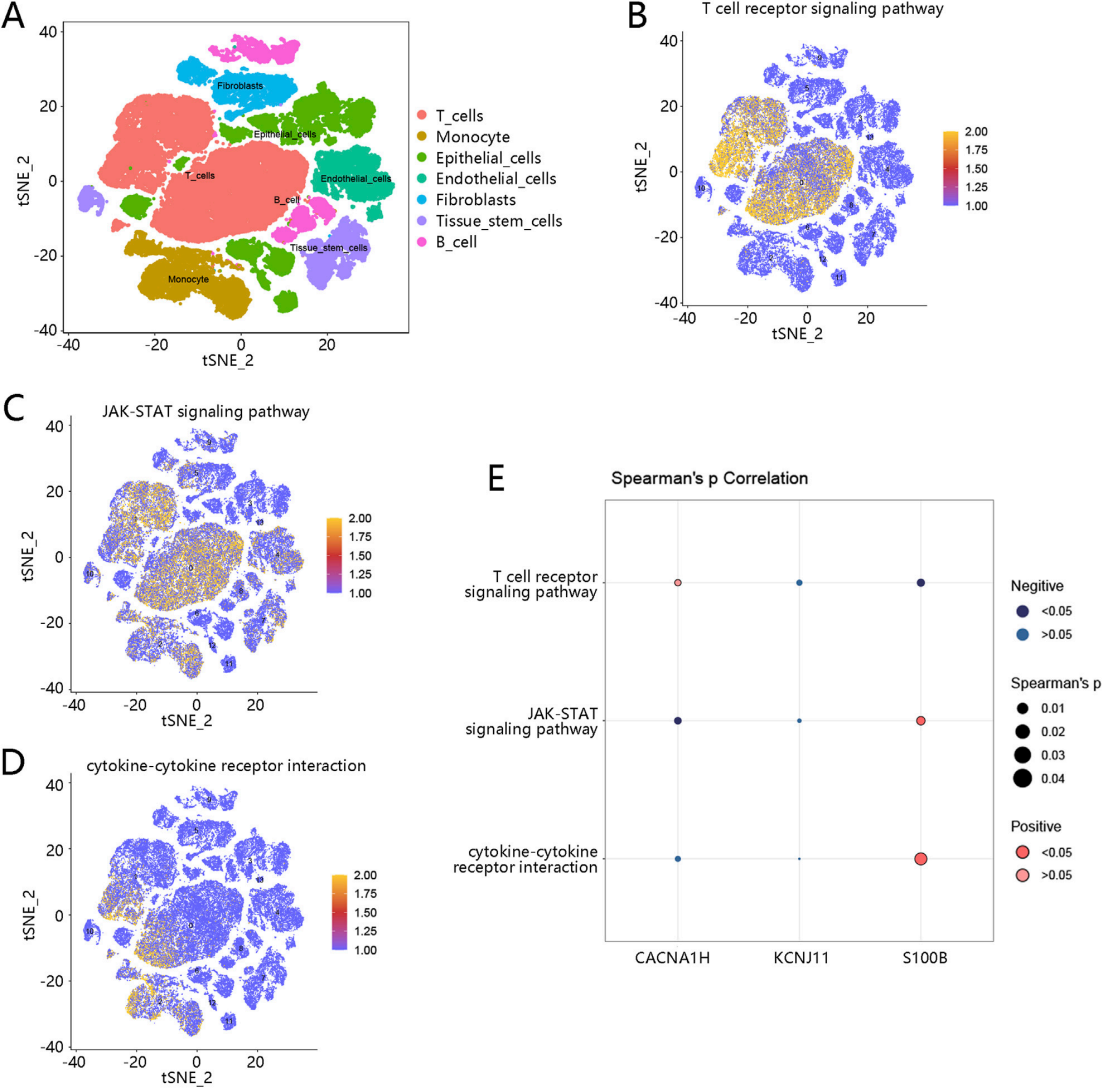
Immune cell distribution and hub gene-related pathways in TNBC **(A)** Seven cell types were annotated based on a single cell-RNA sequencing dataset; different colors represent different cell types. **(B)** Pathway scores of T cell receptor signaling pathway in cells. **(C)** Pathway scores of JAK-STAT signaling pathway in cells. **(D)** Pathway scores of cytokine-cytokine receptor interaction in cells. Orange-colored dots indicate higher pathway activity scores. **(E)** Correlation of three hub genes with three pathways; dark blue dots represent significant negative correlations, while dark red dots represent significant positive correlations. The size of the dots reflects the magnitude of the correlation coefficients. Abbreviation: TNBC, triple-negative breast cancer.

Because of the significant association between hub genes and immune cells, we then used a single-cell RNA sequencing dataset to explore the immune cell distribution in TNBC. A total of 14 cell clusters were identified (Supplementary Figure S4A), and seven cell types were then annotated, including T cells, monocyte, epithelial cells, endothelial cells, fibroblasts, tissue stem cells, and B cells (Figure 8A). In the TNBC samples, T cells, monocyte, fibroblasts, and B cells were the main cell types (Supplementary Figure S4B). The expression of the three hub genes was examined across these cell types. CACNA1H was primarily enriched in tissue stem cells, KCNJ11 was mainly expressed in epithelial cells, and S100B was broadly expressed in the majority of these cell types, particularly in monocytes, T cells, and epithelial cells (Supplementary Figure S4C).

Furthermore, we explored the pathway scores of three pathways (T cell receptor signaling pathway, JAK-STAT signaling pathway, and cytokine-cytokine receptor interaction pathway) related to hub genes in the identified immune cells. As shown in Figures 8B–D, the pathway scores of these three pathways were higher in the T cells, revealing that these pathways may be more active in T cells. The correlation between these three pathways and three hub genes was further analyzed. As shown in Figure 8E, *S100B* was highly associated with the cytokine-cytokine receptor interaction pathway and JAK-STAT signaling pathway.

## 4 Discussion

Accurate diagnosis of TNBC is crucial for guiding treatment decisions and predicting patient prognosis. However, due to the complexity of tumor biology and the limitations of current diagnostic tools, accurately diagnosing TNBC remains a clinical challenge (Yang et al., 2023; Tierno et al., 2023). TNBC is known for its immunogenicity, with nearly half of the cases being immune-excluded, and about 33% are in an immune-inert state (Zhao et al., 2020). Previous studies have shown that T cells are associated with the progression and prognosis of TNBC. In this study, 2,397 DEGs between TNBC and other BRCA subtypes were cross-referenced with T cell-related genes, resulting in 750 overlapping genes. LR analysis and three machine learning algorithms (RF, XGBoost, and AdaBoost) further narrowed these genes down to three key hub genes: *CACNA1H*, *KCNJ11*, and *S100B*, which showed strong performance in diagnosing TNBC from other BRCA subtypes and predicting TNBC prognosis. We found that these genes are closely related to the cytokine-cytokine receptor interaction pathway, JAK-STAT signaling pathway, and T cell receptor signaling pathway.

*CACNA1H* and *KCNJ11* are ion channel-related genes. Although ion channels are traditionally associated with neural functions, their roles in cancer are increasingly recognized. *CACNA1H* is a T-type calcium channel gene that has been shown to influence calcium influx, which is crucial for tumor cell proliferation and metastasis, including in BRCA (Scholl et al., 2015; Mei et al., 2022; Ragab et al., 2022). A previous study suggested that *CACNA1H* might be a potential biomarker for survival and treatment response in specific BRCA subtypes (Pera et al., 2016). *KCNJ11* is a subunit of the ATP-sensitive potassium channel (Lahmann et al., 2019), and mutations in this gene are associated with hyperinsulinemia (Sempoux and Kloppel, 2023). Several studies have also indicated that *KCNJ11* can be used to predict BRCA prognosis (Qiu et al., 2024; Zhu et al., 2021). *S100B* is part of the calcium-binding protein S100 family and acts as an inflammatory mediator. Numerous studies have shown its relevance to BRCA prognosis (Tian et al., 2020; Li et al., 2023). The diagnostic model based on these three hub genes is highly effective, with an AUC value of 0.917 for TNBC, demonstrating strong predictive ability for TNBC. In the prognosis analysis, high expression levels of *CACNA1H* or low expression levels of *KCNJ11* and *S100B* were associated with poorer OS in TNBC patients. Further expression analysis revealed that, compared with normal breast tissue, the expression levels of *CACNA1H* and *KCNJ11* were significantly upregulated in BRCA tissues, whereas *S100B* was markedly downregulated. These findings suggest that *CACNA1H* and *KCNJ11* may function as potential oncogenes, while *S100B* may act as a tumor suppressor. However, in the TNBC subtype, we observed an opposite expression pattern: *CACNA1H* and *KCNJ11* were significantly downregulated compared to other breast cancer subtypes, whereas *S100B* showed markedly elevated expression. This discrepancy in expression patterns may reflect the high degree of molecular heterogeneity present in TNBC. We hypothesize that *CACNA1H* and *KCNJ11* are restricted in their expression within TNBC, being highly expressed only in specific patient subgroups. Single-cell transcriptomic analysis supports this assumption: among the seven major cell types identified, *CACNA1H* expression was primarily observed in tissue stem cells, while *KCNJ11* was mainly expressed in epithelial cells, and both genes showed low expression across other cell types. In contrast, *S100B* was broadly expressed across multiple cell populations, with notably high levels in monocytes, T cells, and epithelial cells. These findings suggest that *S100B* may play a more prominent role in immune regulation and the TME.

In the TME, we found that the diagnostic score was strongly correlated with immune cell infiltration, where lower diagnostic scores were associated with reduced levels of multiple immune cells, including CD8^+^ T cells and cytotoxic lymphocytes. These immune cells are known to be critical in tumor suppression and response to immunotherapy (Farhood et al., 2019), implying that TNBC patients with lower diagnostic scores may experience immune evasion. This finding is consistent with previous studies demonstrating that TNBC is often characterized by an immunosuppressive TME, contributing to poor patient outcomes (Ding et al., 2023). The increased exclusion score in the low diagnostic score group, as demonstrated by the TIDE analysis, further supports this immunosuppressed microenvironment, which is a hallmark of TNBC and a significant barrier to effective immune checkpoint blockade therapy. Our results also revealed that *S100B* was positively associated with CD8 T cells. A previous study reported the expression of *S100B* in CD8^+^ T cells (Houtman et al., 2018). In our research, we found that *S100B* expression was higher in TNBC patients than in other BRCA types. We speculate that *S100B* may serve as a tumor suppressor gene in TNBC, exerting its immune-activating role by activating CD8^+^ T cells. Therefore, single-cell RNA sequencing analysis was performed, which confirmed the significant association between *S100B* and immune cell types in TNBC, particularly T cells. The high expression of S100B in immune cells, especially in T cells and cytotoxic lymphocytes, indicates its crucial role in regulating immune responses within the TNBC.

Additionally, pathway enrichment analysis further highlighted that *S100B* is highly associated with the cytokine-cytokine receptor interaction pathway and JAK-STAT signaling pathway. The cytokine-cytokine receptor interaction pathway plays a critical role in the regulation of the immune system, inflammatory responses, and processes such as cell growth and differentiation (He et al., 2024). After binding to their respective receptors, cytokines trigger conformational changes in the receptors, initiating intracellular signaling cascades, including the JAK-STAT pathway. Wang et al. suggested that immune stemness genes may play a role in lung adenocarcinoma via the cytokine-cytokine receptor interaction/JAK-STAT pathway (Wang et al., 2022). Another study indicated that DEGs, including *S100B*, are associated with the cytokine-cytokine receptor interaction (Qiu et al., 2021). Moreover, RAGE ligands (including *S100B*) can activate the JAK-STAT pathway in rat neurons to promote axonal growth (Saleh et al., 2013). We speculate that *S100B* may regulate the TIME in TNBC via the cytokine-cytokine receptor interaction/JAK-STAT pathway, however, this requires further experimental validation.

This study has several limitations. First, the diagnostic analysis was based on bulk RNA-seq data, which may not fully capture the heterogeneity of TNBC. Second, the top three overlapping genes were identified by multiple machine learning algorithms; although this approach enhances robustness and consistency, it may appear arbitrary and lacks the interpretability provided by methods such as SHAP. Lastly, the proposed mechanism involving S100B and the cytokine/JAK-STAT pathway remains speculative and requires further functional studies. Future research should focus on clinical validation and mechanistic exploration to confirm the translational potential of these findings.

In summary, this study identifies three novel hub genes (*CACNA1H*, *KCNJ11*, and *S100B*) as potential diagnostic and prognostic biomarkers for TNBC. These genes are intricately involved in immune regulation, especially the *S100B*. These findings lay the groundwork for future investigations aimed at integrating molecular biomarkers into precision oncology approaches for TNBC, potentially improving early diagnosis, prognostic stratification, and immunotherapy responsiveness prediction.

## Data Availability

The datasets presented in this study can be found in online repositories. The names of the repository/repositories and accession number(s) can be found in the article/Supplementary Material.
