# Supplementary figures and images for: Identification of three T cell-related genes as diagnostic and prognostic biomarkers for triple-negative breast cancer and exploration of potential mechanisms
Source: Front Genet. 2025 Jun 18;16:1584334. doi: 10.3389/fgene.2025.1584334 (PMC12213735; doi:10.3389/fgene.2025.1584334)

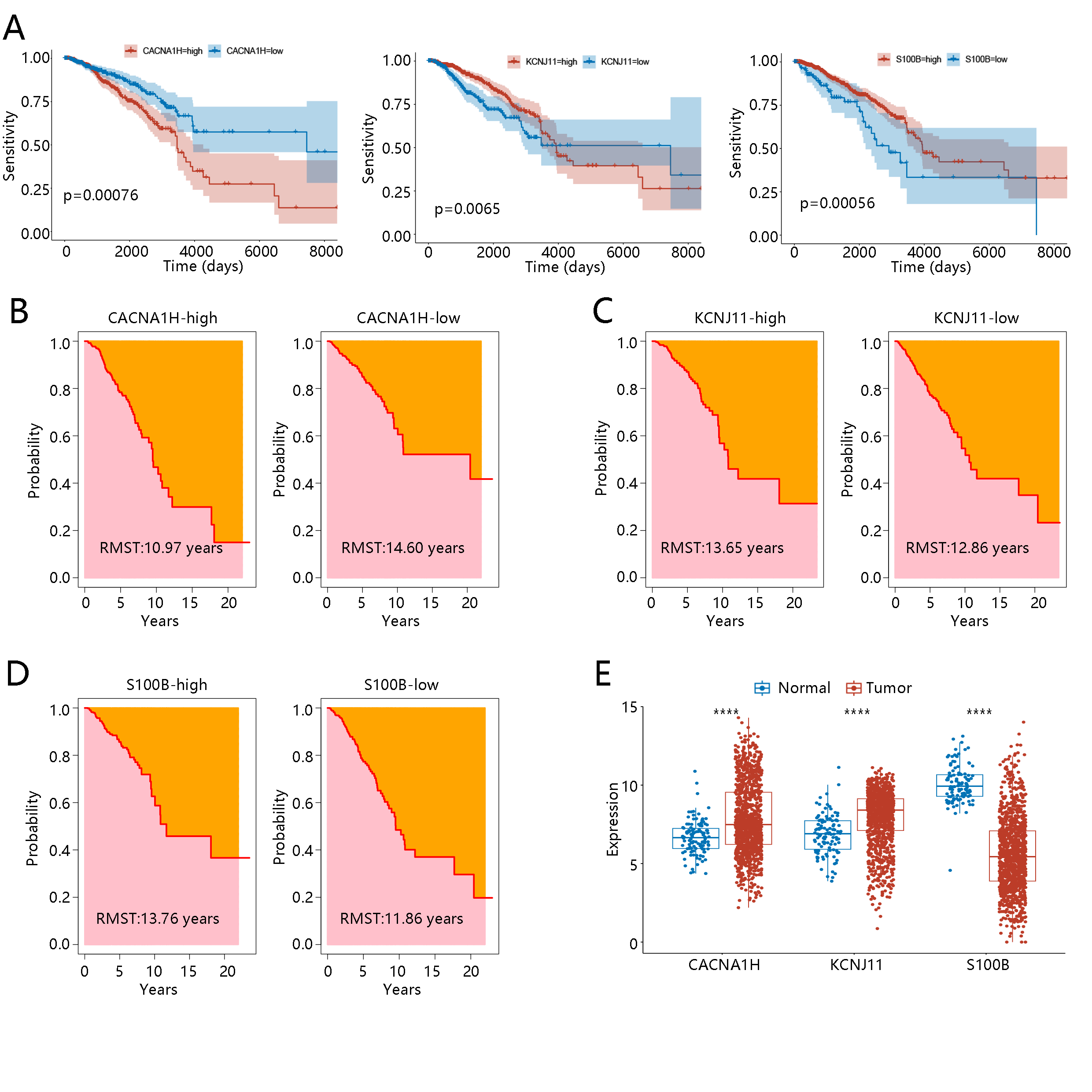

Supplement: Supplementary file 3 [file Image3.tif]

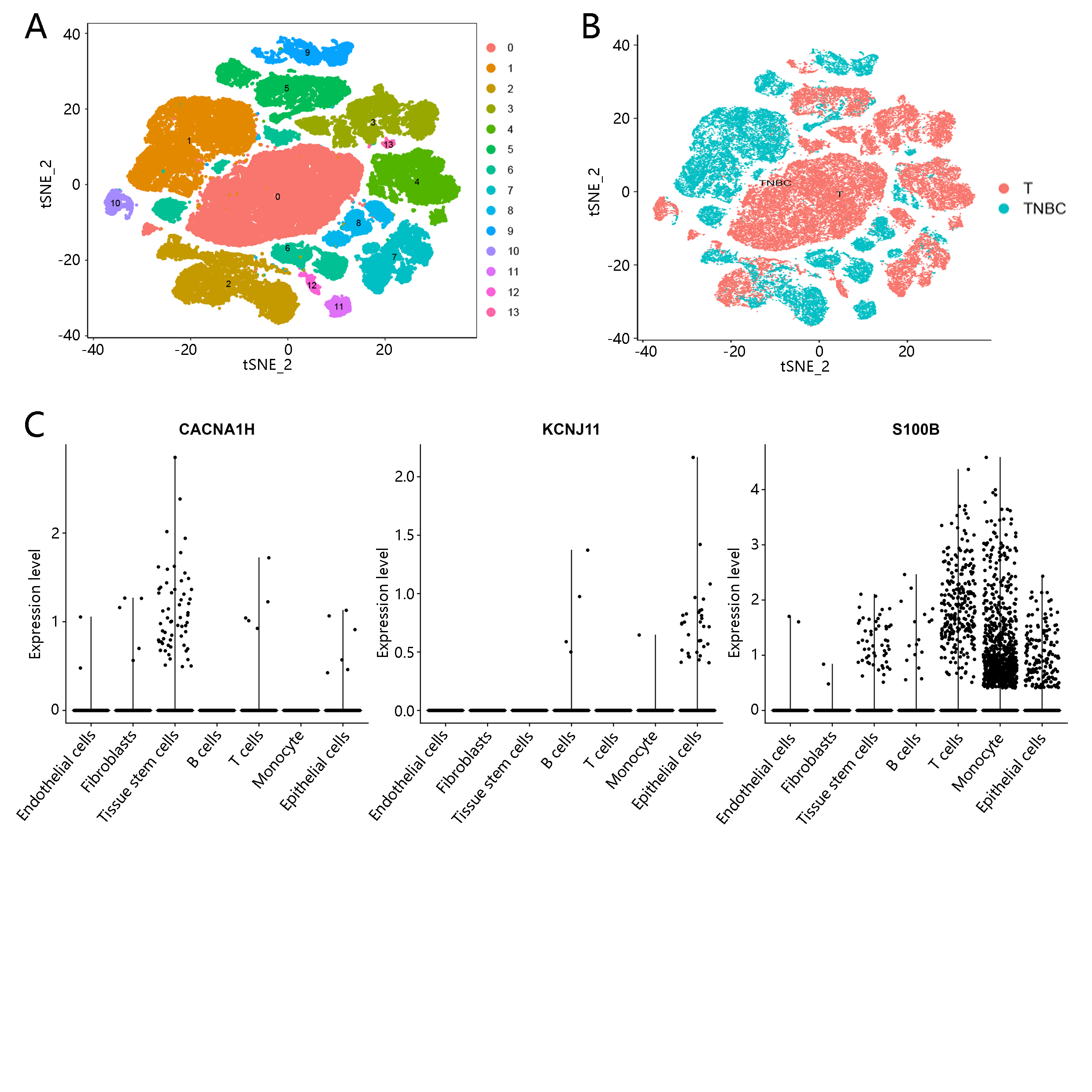

Supplement: Supplementary file 4 [file Image4.tif]

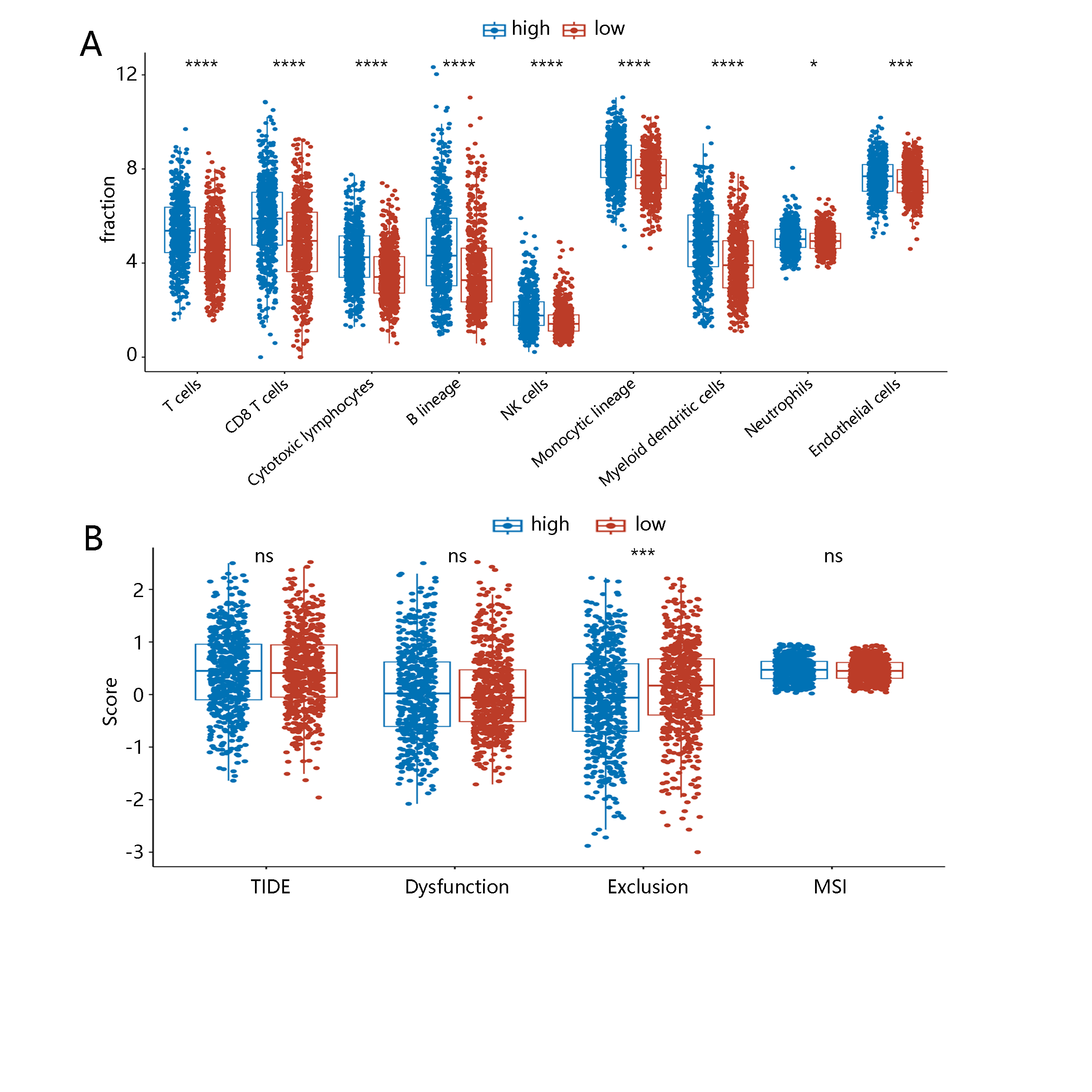

Supplement: Supplementary file 5 [file Image2.tif]

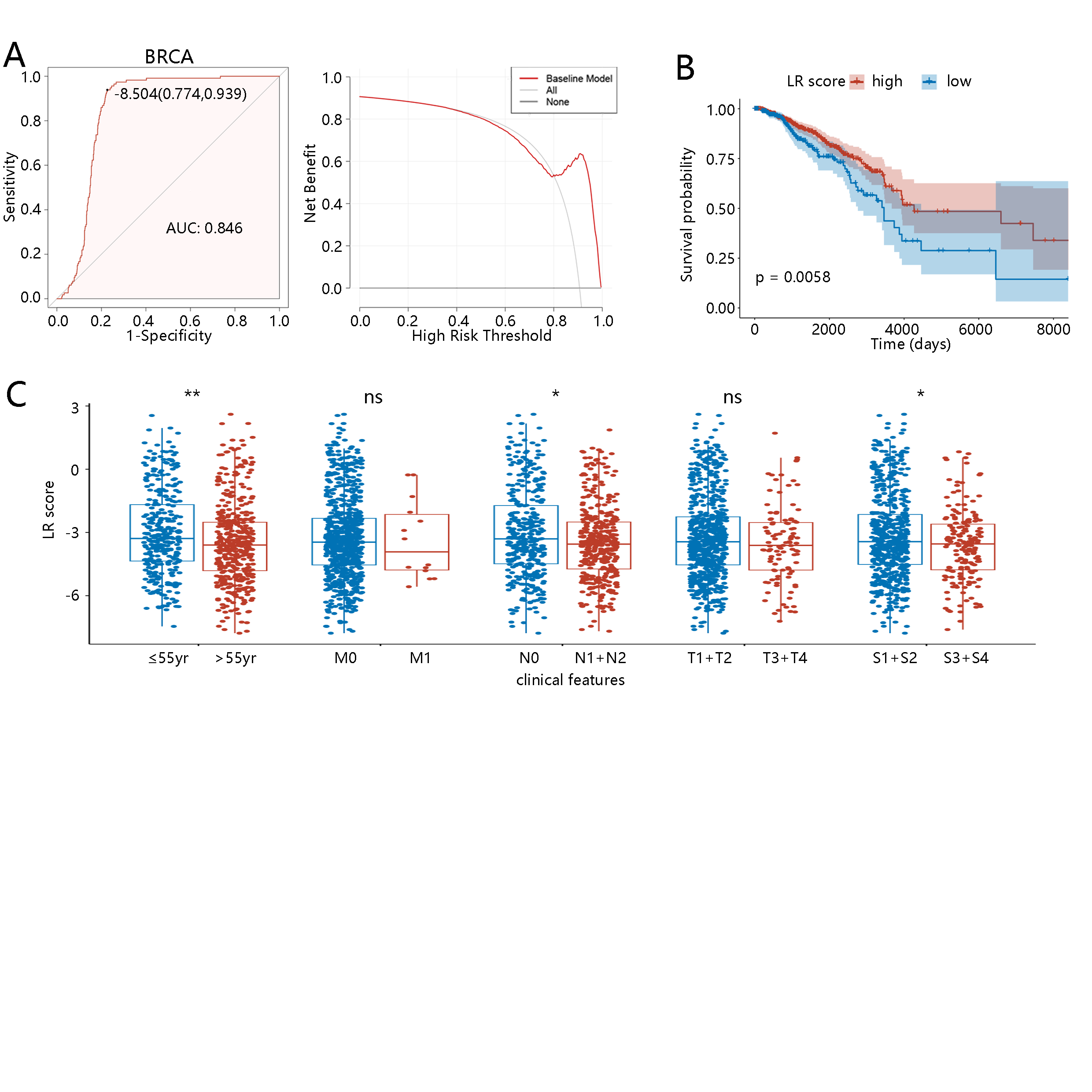

Supplement: Supplementary file 6 [file Image1.tif]
